# Supplementary material for: Are carbon emissions trading and green financial instruments synergistic? -Comprehensive quantitative research based on content analysis
Source: PLoS One. 2024 Mar 7;19(3):e0298601. doi: 10.1371/journal.pone.0298601 (PMC10919601; doi:10.1371/journal.pone.0298601)
Supplement: S1 Data — (ZIP) [file pone.0298601.s001.zip › Data/地级市Z_F工作报告.zip!/╜¡╦╒/┴1⁄4╘╞╕█╩╨/└┤╫╘╣1⁄2╓┌║┼í╛┬φ┐╦╩2╛▌═°í┐_2006.docx]

各位代表：

现在，我代表市人民政府向大会作工作报告，同时根据市委十一五规划基本思路，市政府制定了《连云港市国民经济和社会发展第十一个五年总体规划纲要》草案，请各位代表一并予以审议，并请各位政协委员提出意见。

十五时期和2005年工作回顾

十五时期是奠定新世纪发展基础的重要五年，在中共连云港市委的领导下，全市上下以邓小平理论和三个代表重要思想为指导，全面落实科学发展观，求真务实，开拓创新，克服洪涝灾害和非典疫情等影响，适应宏观环境变化，探索经济发展路子，坚定不移地向富民强市目标迈进，较好完成了

十五确定的主要预期目标，经济社会发展和人民生活迈上新的台阶。

一、结构调整成效明显，经济持续稳定增长

五年来，地区生产总值年均递增11.9%，根据全国第一次经济普查资料核算，预计2005年生产总值达453.9亿元，比上年增长14.2%，三次产业比例调整为20.6∶43.9∶35.5。财政总收入年均递增26.7%，2005年一般预算收入24.6亿元，同口径增长36%。累计完成全社会固定资产投资1110亿元，是九五时期的2.5倍，2005年完成320亿元，增长30%。社会消费品零售总额年均递增11.8%，2005年达165亿元，增长15.3%。国家统计局中心城市综合实力全国百强排名，我市从2001年83位提升到2004年55位。发挥优势培育特色，农村经济健康发展。优化农产品区域布局，水产养殖、高品质粮棉、林木园艺等支柱产业形成一定特色优势，建成全省特色渔业、设施渔业、观光渔业基地，完成造林76万亩，畜牧业良种覆盖率达到80%，东海、灌云均被评为全国绿化模范县、粮食生产百强县。林牧渔业产值比重提高6.9个百分点。农业产业化经营、标准化生产快速推进，省级以上龙头企业发展到16家，农民专业合作经济组织发展到168家，建成无公害、绿色、有机食品品牌130个，四县全部成为省级无公害农产品整体推进示范县。农产品出口创汇位居全省前列。农业综合机械化水平达79%。提升实力壮大规模，工业经济主体地位逐步确立。工业增加值年均递增15.6%，2005年预计达到147.3亿元，规模以上工业利税年均递增21.6%。累计完成全社会工业投资602.4亿元，实施新电公司机组扩建等23项国家技改项目。工业结构逐步优化，新医药、新材料产业形成较强竞争力，纺织机械、电子信息等成长性产业形成一定优势，高新技术产业产值年均递增28.2%，医药、化工、食品等五个支柱行业增加值比重提高到64%。大力推进临港开发和沿东陇海线产业带建设，东方集装箱等临港型项目建成投产。奥神氨纶长丝等3个产品被评为中国名牌，太阳雨热水器等4个产品成为国家免检产品。建筑业快速发展，增加值年均递增15.6%。增强功能优化结构，特色服务业体系初步形成。港口加快向发展要素集聚港转变，现代物流体系不断完善，港口吞吐量连跨4个千万吨级，达到6016万吨，集装箱运量突破100万标箱，开通至韩国客货班轮航线。旅游业快速发展，成功创建中国优秀旅游城市，连岛等4个景区被评为国家AAAA级旅游区，花果山景区被评为全国文明景区，游客接待量和旅游收入分别年均递增18%和20%。成功承办2005年世界旅游日中国主会场暨首届陆桥国际旅游文化节。连续三年成功承办江苏国际农洽会。商贸、餐饮服务业形成集聚发展、品牌经营新格局，成交额过10亿元市场3家。价格总水平基本稳定。开发房地产1250万平方米，商品房销售额年均递增40.1%，新建小区实现物业管理全覆盖，初步建成社区服务网络。信息技术广泛应用，电子政务、电子商务加快推进，电话普及率由14.7%提高到55%，宽带用户发展到9.5万户。金融机构积极支持地方经济建设，信贷有效投入不断扩大，年末各项存款余额437.4亿元，各项贷款余额311亿元，分别比九五末增加236亿元和180亿元。

二、改革开放有序推进，发展活力明显增强

五年来，加快以产权制度为重点的企业改革，市属工业企业、非工业流通企业改革改制面分别达到97.3%和92.4%，国有集体资本基本从中小企业退出，实现资产重组45亿元，妥善安置破产企业职工和离退休人员3.4万人。2家股份公司成功上市，5家公司通过上市辅导验收。民营经济发展迅速，占全市经济比重达49%，比九五末提高31.7个百分点。国有资产监管运营体系日趋完善，组建港口集团、城建等14家资产经营主体，整合组建连云发展集团。政府机构、行政审批和招投标管理体制改革不断深化，探索扩大县级管理权限，实施行政区划调整，撤并云台区和32个乡镇。生产经营型事业单位改制转企加快推进，社会公益型和行政管理型事业单位改革全面启动。财税体制和投融资体制改革有序推进，实施部门预算、国库集中支付和政府采购制度改革，银企银政合作成效明显。

推进重点地区、重点产业和重点项目招商，累计直接利用外资8.8亿美元，引进内联资金270亿元，是九五时期的2.3倍和5倍，2005年实际到帐外资2.6亿美元，同口径增长51%。加快各类园区建设，2005年完成基础设施投入25亿元，园区工业增加值占规模以上工业比重达51%，园区载体功能和集聚效应不断增强。美国杜邦、法国罗盖特等跨国公司落户开发区，出口加工区批准运营。海洋经济开发区、堆沟港化学工业园、浦南开发区等特色园区初具规模，成为县域经济加快发展的重要支撑和主要推动力。外贸进出口总额年均递增31.4%，2005年达19亿美元，外贸出口9.1亿美元，增长20%，地方产品占全部出口的比重达到64%。对外经济技术合作不断扩大，外经营业额和期末在外人数分别年均递增16%和18.6%。

三、基础设施建设加快，城乡环境显著改善

五年来，不断强化基础设施建设，水利、交通、电力、通讯、社会事业等一批重点项目相继实施，地区发展承载能力和服务功能得到增强。实施水库除险加固、海堤达标、河道整治工程，完成一批农村水利建设和城市水环境整治工程。港口新建扩建4个生产性泊位、改造提升14个泊位，扩建了7万吨级航道，实现第五代集装箱船舶全天候进出港，新增综合通过能力1612万吨。连徐、汾灌高速公路全线通车，连盐高速公路完成桥梁路基和路面基层工程，改造国省道495公里，92.6%行政村通水泥柏油路。民航候机楼投入运营，航线航班密度增加。东陇海铁路实施提速改造。海陆空立体交通网络进一步完善，成为交通部规划建设的长三角地区7个国家级综合运输枢纽之一。完成城乡电网建设改造，伊芦、云台等一批输变电工程投入使用。田湾核电站1号机组投料调试。实施电话扩容、连徐光缆、宽带城域网等信息网络基础建设。市急救中心、新图书馆、淮工教学综合楼、省海上训练基地等一批社会公共事业项目投入使用，市新博物馆、少儿活动中心、新闻中心基本建成。

强化城市规划引导，完成城市总体规划修编和市域城镇体系规划，编制港口总体规划、开发区发展概念规划、新海地区分区规划，一批市政设施专项规划相继完成。组织开展东部滨海地区发展战略规划国际竞赛和控制性详细规划。建成区控制性详细规划覆盖率82%。协调推进新区开发和旧城建设，五年投入城市建设资金160亿元，城市建成区面积从51.4平方公里扩展到69.6平方公里。新浦新区建设全面展开，道路网架基本形成，市行政中心主楼建成使用，配套服务设施快速推进。东部城区建设拉开框架，港城大道建成通车，撤除朝阳收费站，改建中山东路，国际商务大厦主体封顶。旧城改造步伐加快，拆除危旧房屋234万平方米，新建住宅509万平方米。新建改建城市道路126条，新增城市道路123公里，新海城区一环、二环路网基本形成。供水、排水、燃气、供热、环卫等公用事业快速发展，大浦污水处理厂投入运行。城市绿化覆盖率达37.3%，人均公共绿地7.5平方米，建成人防工程12.8万平方米。实施城市管理综合执法，初步形成市容环境卫生长效管理机制。开展四城同创，创建国家环保模范城市和省级园林城市通过省级验收。县城建设快速推进，重点中心镇建设加快，市政配套工程同步实施。城乡一体化有序推进，城市化水平由28.1%提高到38.3%。

四、科技教育全面进步，社会事业协调发展

五年来，加快推进高新技术产业发展，实施省以上火炬计划96项、星火计划243项，培育国家高新技术企业9家、省高新技术企业38家，形成海产品养殖加工等8个省级星火支柱产业。建成新医药、硅材料2个国家火炬计划产业基地和中电华威、恒瑞股份2个国家863计划成果产业化基地，成为苏北地区仅有的4个国家级高新技术产业基地。推动企业技术创新和产学研联合，培育5个国家博士后科研工作站、8个省工程技术研究中心，创建留学人员创业园，与中科院、清华大学等建立合作关系。支持中国大陆第一口科学钻探井圆满完成综合测井任务。知识产权保护得到加强，社会科学研究不断推进。完善以县为主的教育管理体制，义务教育成果得到巩固，小学、初中入学率分别达到99.9%和99.7%，对8.2万名农村贫困生实行两免一补政策。创建5所省四星级高中、15所省三星级高中，高中段入学率达87.3%。实施中小学布局调整，全市调整撤并中小学1015所，迁建5所高级中学，新海高中兼并东方中学，教育资源得到优化配置。新扩建中小学校舍197万平方米，改造危旧校舍84万平方米，中小学办学条件明显改善。淮工办学规模不断扩大，地方高校联合初见成效，高校在校生扩大一倍，培养本专科毕业生4万人。职业教育、成人教育长足发展，打破行业限制优化重组10所职业学校，创建省级以上重点职业学校15所、省级农科教示范基地5个。加强人才培养引进，优化人才结构，各类人才总量达到23万人，6人荣获全国劳模和先进工作者称号。加快社会事业进步，促进全面协调发展。文艺创作、新闻出版、广播电视健康活跃，一批文艺作品和广播电视作品获省以上奖。发掘海州双龙汉墓，完成将军崖岩画、孔望山摩崖造像防风化工程。启用数字电视中心大楼，完成中波发射台迁建，全市行政村有线电视联网率达68%。医疗资源有效整合，公共卫生体系建设逐步加强，传染病发病率控制在省平均水平以下，市一院二院分别兼并东方医院和省盐业医院，中医院被评为三级医院。成功举办国际国内沙滩排球和十运会四个项目等重大赛事，获十运会最佳赛区和组织工作贡献奖，五年获省以上金牌182枚。建成全民健身工程28个、健身点252个，常年参加体育活动人口达45%。完成第五次人口普查、第一次经济普查。建成新一代天气雷达、数字化测震台网和省级防震减灾科普基地。外事、侨务、对台工作有效服务经济建设，档案、地方志、老龄、关心下一代事业得到重视发展。

三项国策得到落实，可持续发展能力逐步增强。人口与计划生育管理服务体系不断健全，完善利益导向机制，稳定低生育水平，计划生育率达90%。加强国土资源管理，整顿土地市场秩序，复垦新增耕地5.7万亩。关停整治违法采石塘口，实施云台山封山育林，植被覆盖率达73.9%，五年提高12.5个百分点，海域综合管理水平不断提高。全面推进生态建设，环境质量明显改善，森林覆盖率提高6.5个百分点，市区空气质量优良率88.8%，蔷薇河饮用水源水质达标率保持100%，进入全国污染控制最好城市行列。

五、人民生活不断改善，社会保障逐步加强

五年来，城乡居民收入持续增加，人民生活水平不断提高。城镇居民人均可支配收入年均增长11.2%，农民人均纯收入年均增长7.8%，2005年分别达10006元和3780元，增长12.8%和8%。城乡居民消费结构不断优化，耐用消费品数量迅速增长，汽车等贵重消费品逐步进入家庭，教育文化、休闲娱乐等消费比重大幅提高。城乡居民人均住房面积分别增加6.3和2.1平方米。城市惠民工程得到实施，新建改建便民道路130条，发展管道燃气用户2.3万户，新增公交车90辆。农村实施七件实事，基本完成草危房改造，新增改水受益人口133万人，建成农村公路3800公里，加强农业综合开发，新型农村合作医疗人口覆盖率达90%。实施农村税费改革，取消农业税，农民人均负担下降79%。实施四级联动等帮扶措施，投入帮扶资金11.5亿元，贫困户较九五末减少8.1万户。落实积极的就业政策，强化就业指导服务，建立就业平台，多渠道扩大就业岗位，城镇登记失业率控制在4.5%以内。组织各类职业技术培训98.8万人次，实施劳动力输出百万工程，年新增农村劳动力转移10万人。发放各类就业补贴2603万元，减免税收2654万元，发放小额贷款875万元，政府购买就业岗位2390个，实现年下岗失业人员就业再就业2万人以上。社会保障体系日趋完善，社会保障能力逐步提高。企业职工养老保险覆盖面达98%，离退休人员养老金社会化发放率100%，医疗保险覆盖面提高到88%，失业保险覆盖面达96%，工伤、生育保险范围不断扩展。农村养老保险参保人数达54.5万人。失地农民生活得到基本保障，妥善解决拖欠农民工工资问题。积极开展社会救助，完善城市居民最低生活保障制度，启动农村最低生活保障工程，城乡居民参保人数达11.2万人，残疾人等特困群众基本生活得到保障，改扩建农村敬老院56所，五保对象集中供养率由10.1%提高到35%。

六、精神文明建设不断深入，民主法制建设进程加快

五年来，坚持用正确的舆论导向和丰富的道德实践引导规范市民行为，培育和弘扬新时期连云港精神，营造积极向上、团结奋进的社会氛围，凝聚人心参与改革发展实践。以创建省级文明城市为龙头，整体推进各类文明创建活动，电信公司、柘汪镇被评为全国文明单位和文明村镇，雷锋车成为全国精神文明建设先进典型，抗日山烈士陵园被评为全国青少年教育基地。奥运申吉影响广泛。举办五届在海一方广场文化活动，开展共建文化先进村等活动，城乡文明程度和市民素质稳步提高。自觉接受人大法律监督、政协民主监督和社会舆论监督，注重听取各民主党派、工商联、专家学者和人民团体意见，政府决策机制逐步完善。推进村民自治和社区居民自治，基层民主进一步加强。实施重大事项社会公示、听证制度。完成四五普法任务，构建社会治安大防控体系，依法严厉打击各类违法犯罪活动，平安连云港创建扎实推进。强化行政监察和审计监督，加强政风行风和廉政建设，依法治市有序推进。少数民族扶贫开发成效显著，宗教事务依法有序管理。国防建设、民兵预备役和优抚安置得到加强，两次被评为全国双拥模范城。健全安全生产监管责任体系，安全生产形势进一步好转。开展农资、食品、药品、小化肥等专项打假，市场经济秩序进一步规范。落实信访责任制，完善社会矛盾纠纷大调解机制，妥善处理人民内部矛盾，有效维护了社会稳定。

各位代表，过去的五年，是我市经济实力明显提升、社会事业协调发展的五年，是市场取向改革取得突破、对外开放不断加快的五年，是城乡面貌大为改观、人民生活持续改善的五年。这些成就，凝聚着全市人民的辛劳和智慧，也倾注着方方面面的努力和奋斗。在此，我代表市人民政府，向全市广大干部群众、驻连部队、武警官兵、公安干警、驻连部省属单位和支持连云港发展的海内外朋友，向各位人大代表、政协委员和各民主党派、各人民团体、各界友好人士，表示崇高的敬意和衷心的感谢！

在回顾成绩的同时，我们也清醒地看到，全市发展仍面临不少困难和问题。主要表现为：经济总量较小，财政实力偏弱；产业结构不够合理，经济增长方式需要进一步转变；产业承载能力不够强，带动发展的重大项目比较少；城市规划建设和经营管理水平有待进一步提高，城市化进程比较滞后；县域经济基础还较为薄弱，农民持续增收难度较大；社会保障体系还不够完善，就业工作有待进一步加强；发展环境需要进一步改善，政府工作效能需要进一步提高，行政行为不规范及少数政府工作人员违法违纪现象仍然存在。过去五年的工作实践告诉我们，高度重视和切实解决这些问题，我们必须坚持发展第一要务，更加注重加快发展、科学发展；必须坚持改革创新，更加注重转变观念、创新机制；必须坚持执政为民，更加注重以人为本、富民优先；必须坚持统筹发展，更加注重社会稳定、和谐共存；必须坚持依法行政，更加注重真抓实干、高效廉洁，推动全市经济社会又快又好发展。

十一五时期工作展望

十一五时期是全面建设小康社会的重要时期，是奋力推进我市跨越发展的关键阶段。我们必须进一步增强忧患意识、责任意识、发展意识，抓住难得的战略机遇期，转变发展观念，创新发展模式，提高发展质量，推进我市经济社会发展再上新台阶。十一五时期经济社会发展的总体要求是：以邓小平理论和三个代表重要思想为指导，认真贯彻党的十六届五中全会和省委十届九次全会精神，按照市委九届六次七次全会部署，围绕全面建设小康社会的总体目标，落实以工兴港、以港兴市、以市带农发展方针，以科学发展观统领经济社会发展全局，以跨越发展、致富百姓为目标，以解放思想、开拓创新为动力，坚持工业第一方略，切实加强三农工作，加快建设城市，全面扩大开放，强力推进改革，统筹城乡发展，建设三个文明，构建和谐社会，加快建设国际性海滨城市、现代化港口工业城市和山海相拥的知名旅游城市。全市国民经济和社会发展主要预期目标为：到2010年，地区生产总值突破1000亿元，年均增长16%以上，人均生产总值达到20000元以上，三次产业结构调整为11∶53∶36；财政总收入达到140亿元，其中一般预算收入70亿元，年均增长25%；累计完成全社会固定资产投资2600亿元以上；城市化水平达到48%；城镇居民人均可支配收入年均增长10%以上，农民人均纯收入年均增长8%以上。十一五规划纲要提出的上述目标体现了又快又好的发展要求，既提出积极增长目标，又考虑现实可行性，抓住战略机遇期，充分利用各方面综合条件，实现提速赶超、跨越发展、争先进位；既提出促进经济快速发展，又考虑构建和谐社会，适应时代发展要求，充分体现科学发展观，实现加快发展、科学发展、和谐发展。经过五年的奋斗，努力在以下几个方面取得突破：

实现产业发展新跨越。走新型工业化道路，全力推进产业结构优化升级，做强产业经济，提升综合实力，工业增加值突破450亿元，地区生产总值和财政收入实现翻番，逐步建成沿海工业强市。优化产业发展布局，大力推进沿海大开发和沿东陇海线产业带建设，构筑一纵一横产业走廊，拉开T型产业框架，搭建产业发展平台。突破临港大工业，大力发展石化、能源、造船等产业，隆起沿线制造业，发展壮大硅资源加工、食品加工、机械、现代造纸等产业，形成临港沿线集聚发展、产业经济蓬勃振兴的新局面，建成先进制造业集聚地和区域性重型工业基地。做强做大优势产业，加快产业集聚和企业集群，打造医药、信息材料、化工、食品、机械、纺织、能源等七大产业群。加快发展都市型制造业，着力引办无污染加工业，大力推进高新技术产业化，形成高新技术产业和传统优势产业相融合的现代工业体系。着力构建现代服务体系，优先发展与临港工业和先进制造业配套的生产性服务业，提升金融、信息、物流等综合服务能力，建立高效、开放、畅通、统一的现代商品流通体系，社会消费品零售总额和服务业增加值翻一番以上。

塑造海滨城市新形象。

以先进理念和国际水准规划建设中心城市，构成城区各组团同步发展、山水园林生态融为一体的城市形态，建设空间布局合理、配套设施健全、服务功能完备、环境秩序优良的国际性海滨城市。快速突破东区，加快建设港湾服务区、中央商务区、滨海居住区、产业集聚区，把东部城区建成区域性国际物流中心和国际商务中心、临港产业基地和旅游度假胜地。着力提升新海，加快城市建设改造步伐，增强文化商贸服务功能，提高城市品位和综合服务水平。深度开发山海旅游资源，建设一批高档次高品位的居住生活、休闲娱乐设施，完善城市立体交通体系和信息网络，增强城市现代化水平、综合承载力和对外影响力。建成国家园林城市、国家环保模范城市、国家卫生城市、省级文明城市。2010年中心城市建成区突破100平方公里，人口突破100万，形成沿海中部极具魅力和适宜人居的特色海滨城市和休闲旅游之都。积极发展赣榆，推进赣榆加快融入中心城市组团，形成环海州湾海滨城市三极带动发展态势。突出绿色生态和个性特色，推进东海、灌云、灌南县城向中等城市迈进。加快城市化进程，构建中心城市、县城、重点中心镇协调发展功能互补的城镇体系。

建设社会主义新农村。按照工业反哺农业、城市支持农村的方针和生产发展、生活宽裕、乡风文明、村容整洁、管理民主的要求，坚持从实际出发，以工业化致富农民，以城市化带动农村，以产业化提升农业。构建农民增收长效机制，加大对农业和农村的投入，推进农业科技创新和普及，提高农业综合生产能力，加快建设现代高效农业，大力发展乡村民营经济和小城镇经济，有效推动农村劳动力向二三产业转移，农产品加工率提高到60%以上，农业从业人员比重下降到35%以下。切实增强农村发展活力，完善农村基本经营制度，发展适度规模经营和各类专业合作组织，大力发展农村公共事业，改善农村金融服务，健全村民自治机制，培养有文化、懂技术、会经营的新型农民，实现农民出有所行、学有所教、病有所医、老有所养，对农村学生实行义务教育全免费，行政村全部通客运班车。加快推进县域经济发展，推动县域工业化、城市化、外向化、农业产业化，壮大县域经济整体实力，争取有一个县进入全国百强县行列，100个贫困村实现整村脱贫。逐步形成城乡统筹发展机制，努力走出一条产业互动、城乡融合的建设社会主义新农村的路子。开创对外开放新局面。深入实施互利共赢开放战略，营造有利于投资兴业的政策环境、市场环境和法制环境，全面提升经济国际化水平，在更大范围、更宽领域、更高层次融入国内外区域合作。统筹整合港口、岸线、土地、自然资源等多种发展要素，强力推进新一轮招商引资，着力引进跨国公司、国际财团和国内知名企业，突破一批基地型、带动性大项目，实际到帐外资增长2倍以上，引进国内资金年均200亿元以上。强化园区集聚效应，提高投资强度，构建布局合理、功能配套、特色鲜明的园区体系，市级以上园区工业销售收入、利税、增加值分别翻两番以上。提升外贸外经质量和效益，口岸进出口总额、地方自营出口总额翻一番以上，外经营业额年均增长20%以上。建设区域性国际物流枢纽城市，形成较为完善的现代物流产业体系。发展一体两翼港口群，强化主港区，拓展新港区，加快向规模化、大型化、专业化码头方向发展。加快埒子口、灌河口、海头、柘汪港区基础设施建设，建成带动区域经济发展的组合大港。港口吞吐量1.2亿吨以上、集装箱运量突破400万标箱，显著提升在沿海港口中的地位。全面建成服务周边、辐射中西部的国际商务中心，大力发展商务会展经济，确立在苏北地区对外开放的优势地位。

迈向和谐社会新阶段。把维护好实现好发展好人民群众的根本利益放在第一位，着力解决好群众最关心最直接最现实的利益问题。多渠道增加居民收入，逐步扩大中等收入者比重，提高低收入者收入水平，重视解决弱势群体实际困难，缓解收入分配差距扩大趋势。城镇居民人均可支配收入和农民人均纯收入分别超过16000元和5600元。注重解决城镇新成长劳动力、失业人员再就业、农村劳动力转移问题，社会就业相对充分。优先发展科技教育，协调发展社会事业，建设结构合理、功能完备、开放竞争的区域创新体系，形成基础教育、职业教育、高等教育互促并进的发展格局，营造人才辈出、人尽其才的良好氛围，规划建设高教科技园区、市体育中心、文化艺术中心、新档案馆和传染病医院，逐步形成覆盖全社会的公共服务体系。大力发展循环经济，积极推进循环利用、生态农业、绿色消费，加快建设资源节约型、环境友好型社会，增强可持续发展能力，建设绿色连云港。强化社会保障体系、社会安全体系、社会应急救援体系，逐步确立社会信用体系。扎实推进平安连云港、法治连云港建设，加强和谐社区、和谐村镇、和谐企业创建，提升社会凝聚力和向心力。

2006年工作意见

今年是实施十一五规划开局之年，做好今年的工作，对于落实新一轮跨越发展战略，加快形成大开发、大开放、大发展新局面，具有十分重要的意义。今年政府工作将牢牢把握夯实基础、全面提速两大主题，加大投入、会战东区、重抓环境、力促和谐，确保首战必胜。主要预期目标是：地区生产总值增长16%，财政一般预算收入增长25%，全社会固定资产投资增长25%，社会消费品零售总额增长14%，外贸出口增长16%，实际到帐外资增长30%，居民消费价格涨幅3%，城镇居民人均可支配收入增长10%，农民人均纯收入增长8%以上，城镇登记失业率控制在4%左右，人口自然增长率控制在7‰以内，城市化水平提高2个百分点以上。

全市认真抓好十项重点工程：⑴完成海堤达标防护工程，建设海堤公路。⑵建设连云港国际商务中心。⑶实施新海地区南西入口整治工程。⑷建成机场路、东方大道和花果山北路。⑸建设25万吨级矿石码头和客运码头工程。⑹启动建设港口南港区疏港通道。⑺开工建设市体育中心、高教科技园区，建成市文化艺术中心主体工程。⑻开工建设新沂河50年一遇工程。⑼实施连岛风情区、国际游艇俱乐部、白沙综合娱乐城工程。⑽建设垃圾焚烧热电联产工程。

全市重点做好十件实事：⑴城镇新增下岗失业人员再就业、困难人员就业2.5万人以上，开展各类职业技能培训20万人次。⑵实现农村义务教育阶段学生免收学杂费。⑶城乡居民最低生活保障做到应保尽保，农村五保户集中供养率50%以上。⑷建成经济适用房、拆迁安置房60万平方米。⑸完成40条城市主次干道和便民道路改造，开通新浦至连云地区公交车，建成通村公路800公里。⑹实施中山东路以南旧城改造。⑺墟沟污水处理厂建成使用。⑻有线电视行政村联网率80%以上、入户率达40%。⑼建成市救护总站紧急救援中心。⑽完成48个重点中心镇、一般乡镇总体规划和300个村建设规划，启动50个样板村住宅、基础设施和公共配套设施建设。

为完成上述目标和任务，着重抓好以下五个方面工作：

一、以提升产业整体素质为核心，加快壮大经济综合实力

推进产业结构优化升级，做强工业主体经济。做好沿海开发等产业和空间布局规划，大力推进沿海开发和沿东陇海线产业带建设，加快发展重化工为主的临港产业，发展一批投入产出比高、投资回收期短、上下游产业链长的工业项目。推进50个重点竣工项目如期建成投产，促进30个重点开工项目顺利开工实施，推动浮法玻璃二期、益海油化项目等一批投资规模大、拉动效应明显的重点项目建设，实施杜钟氨纶等一批重点技改项目，完成工业投资210亿元以上，100个新增长点项目对工业增长贡献率70%以上。引导优势产业扩大规模效应、提升核心竞争力，推动45户销售收入过亿元企业规模扩张，争取有7户企业营业收入过10亿元。进一步培育壮大建筑业，完成建筑业总产值120亿元。按照高产、优质、高效、生态、外向、安全现代农业发展定位，加快调整农业产业结构。大力培育农业主导产业和县域规模特色农业，强化优质稻米、高品质棉等主导品牌，提升林业经济效益，扩大特色渔业和水产品加工业，发展畜牧业规模养殖，提高农业综合效益和竞争能力。推进农产品质量安全标准体系建设，建设生态农业县，增强重大动植物疫病防治能力，新增农产品品牌10个。推进园艺、蔬菜、海产品等出口基地建设，扩大外向品牌效应，建设沿东陇海线农产品出口示范区。重点发展一批都市生态观光、农村田野风情和休闲渔业项目。积极推进产业化经营，重点扶持16家省级以上和37家市级农业龙头企业。提高市场化组织程度，发展20家农民专业合作经济组织，建立市级农牧业生产、经营、出口协会，加强农村经纪人队伍建设，增强农产品进入市场能力。强化农业科技人员队伍建设和农业科技培训，开展科技入户工程、阳光培训工程，提高农业科技水平。稳步加大农业投入，加快农业综合开发，推进现代流通业态下乡，加强农村生产服务体系和金融服务体系建设，落实税费减免政策，促进农民增收农业增效。

提升功能打造特色，加快发展现代服务业。加快建设以港口为龙头的现代物流中心，强化口岸大通关建设，完善港口集疏运功能，增加远洋和内贸航线航班，推进中云物流园区和货物交易中心建设。完成港口吞吐量7000万吨，集装箱运量130万标箱，力争150万标箱。加快推进空港建设，增辟新的航线航班，争取开放航空口岸。整合旅游资源，申报成立花果山影视基地，建设旅游大厦、花果山游客服务中心和云台山数字化风景区，组织连云港旅游万里行活动，进一步打造旅游城市品牌。国内外游客接待量突破770万人次，实现旅游总收入67亿元。加快发展以专业市场、连锁经营、电子商务为支撑的商贸流通业，重点培育主业突出、竞争力强的大型连锁企业，社会消费品零售总额188亿元。完善房地产市场体系，促进房地产业健康发展，开工建设住宅300万平方米。推进社区服务体系建设，配套完善社区服务网点，新增小区物业管理覆盖面积100万平方米以上。加快数字连云港建设，加快开发口岸、物流、地理等信息系统，大力发展信息服务业。注重培育发展商务会展经济，积极承办各类重大节庆经贸活动。建设苏北烟草物流中心。培育商务服务机构，加快发展法律会计、咨询评估、广告策划等中介服务业。推进公共财政建设，提高财政支撑保障地方发展能力。建设金融生态环境，完善银行信贷和银企合作制度，扩大有效信贷投放，促进保险创新服务，增强金融服务地方发展功能。

二、以激发经济发展活力为取向，加快推进改革开放步伐

深化和完善企业改革，推进企业资产重组和破产企业土地、财产变现，加快破产企业职工清偿安置，实施改革发展项目12个，实现资产重组2亿元。进一步整合提高国有资产运作能力，发挥连云发展集团集聚效应。加快发展民营经济，培育壮大科技型、外向型、就业型骨干企业，启动微小企业成长计划，开展创业辅导服务，重点培育10个发展前景好的特色产业，新注册私营个体企业注册资本增长20%以上。注重解决中小企业融资难问题，重点扶持一批骨干担保机构，开展民营企业信用评级工作。积极组建完善行业协会、商会，以市场为导向，实行行业服务和自律管理，推进政会分开，促进健康发展。加快事业单位改革，全面完成生产经营型事业单位改制转企，推进社会公益型事业单位人事分配制度改革，归并整合、精简规范行政管理型事业单位。深化财税管理体制改革，规范税收征管秩序，加强非税收入管理，推进国库集中支付，完善市、区财政管理体制。

强力推进新一轮招商引资，继续突出日韩、港台地区，充分开拓欧美、东南亚市场，重点抓好南方资本和环渤海湾地区资本的转移承接，实际到帐外资3.4亿美元，内联到位资金190亿元。依托产业布局调整招引重大项目，加大基础设施投入，力争在谈的码头建设等重大项目取得实质性进展。依托产业转移开展招商引资，创新园区合作模式，增强产业承接能力，力争突破一批产业转移项目。依托自身优势开展招商引资，发挥现有产业基础和资源优势，提高市场化资本运作水平，开展新能源、硅资源、农副产品加工等领域的产业链招商，努力招引一批见效快、带动力强的龙头基地型项目。结合东部城区加快发展、一体两翼组合港建设，科学规划各类园区，在柘汪、海头、燕尾港和灌河口一线规划建设临港产业区。加强园区规模化、特色化建设，着力提升东海、海州等都市型工业园，推进昆山工业园、江宁工业园、上海奉城工业园建设，充分发挥示范带动作用。推进沿海临港各产业区道路建设，加快污水处理、供热等配套设施建设，积极申报保税物流园区。增强园区要素集聚和产业承载能力，构建以国家级开发区为龙头、省级开发区为主体、中心镇园区为基础的园区群。优化出口产品结构，扶持和培育特色出口商品，引导企业扩大自主品牌产品、机电产品和高新技术产品出口份额，提高出口商品的技术含量和质量档次。壮大外贸出口主体，着力引进高附加值、高新技术、高创汇的加工贸易项目，建立一批规模化大宗农副产品出口基地，完成外贸出口10.5亿美元。广泛开展外经合作，推进金海公司境外投资项目建设，支持优势企业参与国际市场竞争，引导有对外承包权的企业提高劳务合作层次。

三、以构建国际海滨城市为龙头，加快提升中心城市功能

围绕凸显国际性海滨城市个性特色，进一步完善城市规划体系，编制近期建设规划。拓展滨海地区发展战略规划成果，加快控制性详细规划编制，抓好城区出入口、干道两侧重点地段的布局规划和单体设计。修编乡镇总体规划，编制村庄建设规划。实行城市规划公示制度，提高规划的透明度和决策水平。健全规划法规体系，增强规划指导、协调、控制能力，强化违法建设查处。

以提升港口综合功能、配套临港产业发展、拓展海滨城市空间为重点，全面掀起东部城区和基础设施会战热潮。按照延伸一体、扩展两翼发展思路，启动主体港区功能调整和陆域整合，加快港口基础设施和配套项目建设，加快推进15万吨级航道扩建，开工建设庙三突堤集装箱码头、墟沟二期5万吨级通用泊位、墟沟三期焦炭码头，加快推进10万吨级氧化铝泊位围堰工程。构筑港口集疏运快速通道和基础支撑体系。规划扩建云台山隧道。实施东陇海线电气化改造，推进客运东站建设和连盐铁路前期工作。启动建设连临高速公路，续建323、236省道。做好海河联运前期工作。实施一批河道整治和水库除险加固工程。建设包庄、虎山、龙尾等一批输变电工程。全面推进东部城区建设，启动海滨新城抛填工程，加快西墅海滨新城建设，完成大港路、中山路、海棠路环境改造，加大旧城拆迁力度，抓好平山花园、滨海花园等安置小区建设。

完善新海城区路网，加快建设改造凌州路、人民路、新建路等城市主次干道。加强城区拆迁整治，推进新华广场、龙河广场周边地块开发改造，开发建设凤凰新城。加快园林绿化项目建设，实施一批道路、公园、滨河重点绿化工程，推进企业、校园、小区园林式建设，新增绿地150公顷。西气东输工程投入使用，发展天然气用户6000户。加快城区污水收集管网建设。深入开展四城同创活动。注重加强城市细节管理，提高城市品位，贴近群众生活。加强市容环境综合整治，对重点地段市容环境实施精细管理。加快平改坡步伐，美化亮化市容，实施亮化项目35项。净化环境卫生，新建改建公厕、垃圾房等一批环卫设施，实行垃圾资源化无害化处置，推进城管进社区，减少城区环境卫生死角。

四、以提高人民生活质量为目标，加快推进社会和谐发展

促进社会和谐发展，从提高市民综合素质和现代文明程度出发，更加注重社会全面进步，协调发展社会事业，实现经济社会可持续发展，积极为人民办实事办好事，进一步改善人民生活条件，保障人民安居乐业，加快构建和谐社会。

努力把科教兴市战略落到实处，提高全社会科学文化素质，增强发展智力支撑。加快推进高新技术产业化，围绕做强星火支柱产业，组织实施火炬计划20项、星火计划50项，高新技术产业产值突破75亿元。强化企业自主创新能力，着力构筑区域科技创新体系。加强农业科技创新，实施农业科技攻关和成果推广计划20项。大力推广应用新技术、新成果，提高经济社会发展的科技含量。坚持教育优先发展，积极推进教育现代化，进一步提高全民受教育程度。完成农村中小学六有工程，推进校校通工程，新扩建中小学校舍10万平方米。加强高校硬件设施建设，提升高校办学层次，推进地方高校申本工作，增强高等教育对地方经济助推作用。大力发展职业教育，扶持重点职业技术院校提高办学质量和水平，积极发展继续教育、网络教育，延长新增劳动力受教育年限。实施人才强市战略，注重抓好公共管理、企业管理、专业技术和实用人才队伍建设，创造条件筹建境外人才培训基地，引进本科以上人才1600人。统筹兼顾发展各项社会事业，不断丰富人民群众精神文化生活，推进社会可持续发展，创造完善舒适的生活环境和清新自然的生态环境。创作一批具有地方特色的优秀文艺作品，加强基层文化建设，健全公共文化服务体系，推进文化市场有序发展，启动建设老年大学。继续推进有线电视进村入户。建设市二院东院区综合楼、传染病院一期工程，加快医疗救治、疾病控制和卫生监督体系建设，城市社区卫生服务中心建成率80%。全面构建群众体育服务体系，认真备战省十六届运动会。积极应对第四次生育高峰，稳定低生育水平，努力遏止出生人口性别比升高势头，计划生育率90%以上。坚持资源开发节约并重，发展循环经济，大力推进节能节水节材和清洁生产，开发风能、太阳能等再生能源，发展农村沼气3000户。完成土地总体利用规划修编，建立土地节约利用机制，落实海洋功能区划。编制实施生态市建设总体规划，切实保护好自然生态，加强矿山塘口植被恢复，加快创建国家环保模范城市，四县全部通过生态示范区验收。开展农业普查，着手地方志续编。抓好外事、侨务和对台事务，做好气象、防震减灾、新闻出版工作，重视发展妇女、儿童和关心下一代事业。

推进精神文明和民主法制建设，创造健康向上的人文环境和安定有序的法制环境。扎实开展群众性精神文明创建活动，全面提高市民道德水准和城乡文明程度，健全社会信用体系，推进学习型城市建设，着力营造讲求实干、崇尚先进的社会风气和互帮互助、融洽和谐的人际关系。定期向人大及其常委会报告工作，坚持重大问题与人民政协协商，认真听取各民主党派、工商联、无党派人士和工会、共青团、妇联等人民团体的意见。积极办理人大代表建议和政协委员提案。健全社情民意反映制度。扩大村民自治、社区自治。启动

五五普法，推动法治连云港建设，增强全社会法律意识，尊重和保障人权。加强社会治安综合治理，严厉打击各类犯罪活动，扎实推进平安创建，努力维护社会稳定，建设市应急指挥中心。做好新时期民族工作，加强宗教事务管理，恢复重建海清寺。加强国防教育和国防动员，搞好拥军优属和军民共建，争创全国双拥模范城五连冠，建成人防应急指挥中心。规范市场经济秩序，强化农资、食品、药品监管，落实安全生产责任制，防止重特大事故发生，提高突发公共事件应急救援能力，保障人民群众生命财产安全。认真做好信访工作，完善社会矛盾调解机制，畅通民众诉求渠道，强化法律援助和法律服务，妥善处理新时期人民内部矛盾。

把富民优先放在头等重要位置，注重解决群众增收、就业、保障等实际问题，让改革发展成果惠及全体人民群众，切实维护和促进社会公平。多渠道增加城乡居民收入，提高经营性、资产性收入在城乡居民收入中的比重，引导企业建立在岗职工收入随效益增长机制，提高最低生活保障和最低工资标准。多层次改善群众生活条件，做好失地农民补偿、农村税费减免、城市动迁居民安置等工作。推进整村扶贫开发，加快老区开发建设。关爱弱势群体，重视解决困难群众住房、医疗和子女就学问题，抓好老龄和残疾人事业。继续落实积极的就业政策，大力发展就业容量大的劳动密集型产业、服务业和中小企业，不断开发就业岗位。努力创造公平公正的就业环境，通过增加培训经费、购买岗位、就业补贴、税费减免等形式，帮助困难群众就业。健全社会保险、社会救助、社会福利和慈善事业相衔接的社会保障体系，完善城镇职工养老、医疗、失业、工伤和生育保险制度，实行

五保合一征缴模式。稳妥推进农村社会养老保险，新型农村合作医疗覆盖面保持在90%以上。增加财政投入，多渠道筹措社会保障基金。

五、以提高依法行政水平为重点，加快提高政府行政能力

面临跨越发展的特殊阶段和弥足珍贵的发展机遇，进一步确立以人为本、执政为民的理念，加强自身建设，提高行政能力，努力做到为人民服务，对人民负责，受人民监督，让人民满意。

牢记宗旨，为民行政。牢固确立全心全意为人民服务的观念，确立科学发展观和正确政绩观。带着责任抓学习，不断更新知识结构，提升思维层次，提高处理各类复杂问题的能力。带着使命促发展，本领用在干事业上，功夫下在抓落实上，全身心地投入工作，加快推进连云港的跨越发展。带着感情帮民富，以工作实效实现人民群众期盼，确保人民生活水平进一步提高。

创新管理，高效行政。把转变政府职能和创新管理方式结合起来，加强社会管理与公共服务职能，该管的切实管住管好，不该管的坚决交给企业、中介组织和市场。继续深化行政审批制度改革，推进职能部门审批机制创新。大力改进机关作风，认真解决条块分割、权责脱节、推诿扯皮等突出问题，加强电子政务建设，提高行政效率，降低行政成本，使机关运转更加规范协调、办事更加公平公正、工作更加高效便民。

科学决策，依法行政。把依法行政贯穿于政府决策、执行、监督的全过程。完善重大问题集体决策、专家咨询、社会公示听证、决策效果评估制度，使决策更加切合实际，更加符合群众利益。健全行政执法体制，规范行政执法行为，加强行政执法监督，严格行政执法责任制度和过错追究制度，促进严格执法、公正执法、文明执法，全面提高依法行政水平。继续推进政务公开，使政府工作更加阳光透明。

严格自律，廉洁行政。坚持从落实制度抓起，建立健全教育、制度、监督并重的惩治和预防腐败体系，认真落实党风廉政建设责任制，充分发挥行政监察和审计监督职能，加强招投标中心、行政效能投诉中心建设，强化权力制约。坚持从逐级监督抓起，把法律监督、民主监督、舆论监督和群众监督结合起来，形成监督合力。加大对违法违纪问题的查处力度，以清正廉洁取信于民。

各位代表，今后五年的发展任务艰巨而光荣，崭新一年的政府工作繁重而紧迫。让我们紧密团结在以胡锦涛为总书记的党中央周围，高举邓小平理论伟大旗帜，认真实践三个代表重要思想，全面落实科学发展观，在中共连云港市委的领导下，团结和依靠全市人民，抢抓机遇，奋勇争先，开拓创新，扎实工作，为开创连云港跨越发展、科学发展、和谐发展的新局面而努力奋斗！
